# The aptitude of commercial yeast strains for lowering the ethanol content of wine

**DOI:** 10.1002/fsn3.1433

**Published:** 2020-02-03

**Authors:** Vladimir S. Puškaš, Uroš D. Miljić, Jovana J. Djuran, Vesna M. Vučurović

**Affiliations:** ^1^ Faculty of Technology Novi Sad University of Novi Sad Novi Sad Serbia

**Keywords:** commercial yeast strains, global warming, lower‐alcohol wines, sequential inoculation

## Abstract

The high alcohol content in wine usually has a negative impact on its sensory properties, but can also affect the general health of the consumers. The possibility to reduce ethanol production in wines during fermentation involves the use of different yeast strains characterized by the increased production of fermentation by‐products (glycerol, 2,3‐butanediol, etc.) from the available sugar. The activity of these strains should not impair the sensory properties of the wine. In general, the use of genetically and evolutionarily (non‐GM) engineered *Saccharomyces cerevisiae* strains is still not close enough to commercial application, and therefore, it is unavailable for wine producers. Thus, the aim of this study was to examine the possibility of reducing the production of ethanol in wines using different selected yeast strains (*S. cerevisiae, Saccharomyces bayanus, Torulaspora delbrueckii,* and *Metschnikowia pulcherrima*) available at the market. The application of individual yeast and sequential inoculation for wine alcoholic fermentation was examined. The achieved effects were evaluated by determining the content of ethanol, as well as fermentation by‐products (glycerol and volatile acids) and aromatic components in wine samples. Depending on the strain/s used, a decrease in ethanol content of up to 0.9% v/v was recorded in comparison with fermentation by *S. cerevisiae* alone. The sensory analysis of produced wine showed significant differences in taste and flavor. The results of the experiment conducted at the laboratory level and with the use of sterile must were compared to the ones from the scale‐up experiment in real vinification conditions. The observed differences in the alcohol content of produced wines were significantly lower.

## Graphical abstract

From the standpoint of consumers, high alcohol content has a negative impact on the sensory properties and human health. The aim of this study was to examine the possibility of reducing the production of ethanol in wines using different selected yeast strains (*Saccharomyces cerevisiae*, *Saccharomyces bayanus*, *Torulaspora delbrueckii*, and *Metschnikowia pulcherrima*) available at the market.

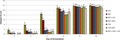

## 1 INTRODUCTION

Climate change, new market trends, and technologies' progress nowadays strongly affect the wine industry. Over recent decades, the average alcohol content of table wines has increased by about 2% (v/v), due to the high sugar content of the grapes currently used in the most wine‐growing regions (Goold et al., 2017). A wide range of factors significantly affects sugar accumulation in the grape such as warm climate conditions combined with the lengthy maturation periods used to satisfy the consumer demand for rich and ripe fruit flavor in the wine. The high alcohol content in wine has several negative consequences. One of the major issues of higher alcohol content in wines is its effect on the sensory properties, which increases the perception of the heat and alters the perception of wine aroma complexity (Goldner, Zamora, Di Leo Lira, Gianninoto, & Bandoni, 2009). Also, excessive alcohol intake through the consumption of wines with higher ethanol levels is often associated with undesirable implications on human health. Furthermore, higher alcohol in wine may increase costs in countries where taxes are levied according to alcohol concentration. Thus, the combination of quality, health, and economic issues associated with high‐alcohol wines has created significant interest in the development of technologies for the production of reduced ethanol wines.

Different approaches to reduce alcohol levels in wines have been proposed at all stages of the winemaking process. These mainly fit into four basic groups as viticultural, prefermentation, fermentation, and postfermentation strategies (Varela et al., 2015). Viticulture strategies, as promising but long‐term techniques, are based on the selection of new grape varieties with low sugar accumulation, viticultural practices adapted to unripe grapes, and different agronomical methods (Olego et al., 2016). On the other hand, postfermentation strategies such as reverse osmosis, nanofiltration, and distillation represent a short‐term perspective dependent on the current EU and OIV regulations. Moreover, these procedures may increase production costs and also can compromise the wine organoleptic quality due to the elimination of volatile compounds (Schmidtke, Blackman, & Agboola, 2012). Considering possible approaches, the application of yeast strains characterized by lower sugar‐to‐ethanol transformation rates has been imposed as an attractive way to deal with the problem of high‐alcohol wines (Kutyna, Varela, Henschke, Chambers, & Stanley, 2010). Lower ethanol‐producing yeast strains could be isolated and characterized from spontaneous wine alcoholic fermentations or obtained through the application of adaptive evolution (development of the low‐alcohol variants of existing *Saccharomyces cerevisiae* strains) and genetic modification techniques (GMO approaches) (Ozturk & Anli, 2014; Varela et al., 2015). Due to poor consumer acceptance of GMO foods and beverages, there is a need to investigate and develop the non‐GMO approaches for the generation of wine yeasts that produce less ethanol (Kutyna et al., 2010).

Nonconventional yeasts such as *Kloeckera*, *Pichia, Candida, Metschnikowia, Schizosaccharomyces,* and *Torulaspora* species are among the main representatives of grape natural microbiota. In general, their pronounced sensitivity to antimicrobial agents (e.g., SO_2_) and higher alcohol contents prevent the complete transformation of grape sugars into ethanol during alcoholic fermentation. Therefore, their application in co‐inoculation or sequential inoculation with *S. cerevisiae* is increasingly getting popular especially regarding their potential positive effects on wine flavor (Ciani et al., 2016). On the other hand, species, such as *Saccharomyces bayanus*, are associated with spontaneous fermentation of must and have been shown to be of oenological interest (González, Barrio, Gafner, & Querol, 2006). The use of mixed cultures of selected *Saccharomyces* and non‐*Saccharomyces* yeasts for wine fermentation can result in the formation of higher amounts of undesired compounds (e.g., volatile phenols and ethyl acetate) which can affect both structure and the aromatic profile of the wines. Therefore, ensuring the expression of appropriate metabolic characteristics of different yeast strains individually or in mixed cultures might serve as an efficient mechanism for reducing ethanol production in wines (Ciani & Comitini, 2015). The application of non‐*Saccharomyces* species for decreased alcohol production is possible through both aerobic (respiration) and anaerobic (fermentation) metabolism (Ciani et al., 2016).

The aim of this research was to examine the possibility of reducing ethanol production in wines using different selected yeast strains (*Saccharomyces cerevisiae, Saccharomyces bayanus, Torulaspora delbrueckii*, and *Metschnikowia pulcherrima*) which are currently available at the market. The experiments implied a series of wine fermentations carried out both by the activity of chosen strains individually and in the form of mixed cultures applied through sequential inoculation.

## 2 MATERIALS AND METHODS

### 2.1 Inoculation strategies for the fermentation of experimental wines

In order to investigate the possibility of reducing the production of ethanol in wine using different yeasts as producing microorganisms, the following commercial strains were used:

1. *Saccharomyces cerevisiae* Oenoferm Bouquet (Erbslöh, Germany), shortened **CER.**
2. *Saccharomyces bayanus* LittoLevure CHA (Erbslöh, Germany), shortened **BAY.**
3. *Torulaspora delbrueckii* Oenoferm Wild and Pure (Erbslöh, Germany), shortened **TOR.**
4. *Metschnikowia pulcherrima* FLAVIA MP346 (Lallemand, France), shortened **MET.**

The yeast inoculation was carried out according to the plan shown in Table 1, for both laboratory and scale‐up experiments. Production of experimental wines was carried out under identical conditions for all samples (laboratory and scale‐up level) and was done in triplicate.

**Table 1:** Inoculation plan the fermentation of experimental wines

Experimental sample	Plan of inoculation with selected strains
CER	*Saccharomyces cerevisiae*
BAY	*Saccharomyces bayanus*
MET+CER	*Metschnikowia pulcherrima* and then 48 hr after the first inoculation of *S. cerevisiae*
MET+BAY	*Metschnikowia pulcherrima* and then 48 hr after the first inoculation of *S. bayanus*
TOR	*Torulaspora delbrueckii*
TOR+BAY	*Torulaspora delbrueckii* and then 48 hr after the first inoculation of *S. bayanus*
MET+BAY+CER	*Metschnikowia pulcherrima*, then 48 hr after the first inoculation of *S. bayanus*, and then 96 hr after the first inoculation of *S. cerevisiae*

### 2.2 Laboratory‐scale experiment

Wines were produced from the Serbian white grape variety Sila (*Vitis vinifera* L.), from grapes originating from the experimental vineyards of the Faculty of Agriculture, University of Novi Sad, located in Sremski Karlovci. Grapes were harvested at the stage of technological maturity (optimal ratio between sugar and acids, phenolic and aromatic maturity also ensured), at the end of September 2016. The processing of grapes included crushing and destemming (Zambelli Gamma 30), followed by pressing in classical vertical basket press (capacity 100 kg). The sugar content in the must was 19.5%, total acidity 5.1 g/L (as tartaric acid), and assimilable nitrogen 225 mg/L. The must was sulfited by the addition of potassium metabisulfite (0.1 g/L) in order to prevent oxidation. The clarification of must was carried out by classic precipitation with the addition of the Trenolin FastFlow pectolytic enzyme (Erbslöh) in the amount of 5 ml/hl. The must was pasteurized (Grant instruments, 75°C during 15 min) to neutralize the influence of autochthonous yeasts on the experimental fermentations. Fermentation was carried out in 10‐liter glass vessels. Must samples were inoculated with selected yeasts and rehydrated according to the manufacturer's instructions, and in the amounts suggested. The yeast nutrient VitaFerm Ultra (Erbslöh) was added after one‐third of sugar fermented (sugar content 120 g/L), in the amount of 0.2 g/L. The fermentation temperature was maintained at 16–18°C. During alcoholic fermentation, several parameters were evaluated in duplicate: sugars, glycerol, volatile acidity, and ethanol. After the end of fermentation, the wines were racked, sulfited (0.08 g/L potassium metabisulfite), and stored in 2‐L glass bottles. The analysis of the major volatile compounds and the sensory analysis of produced wines were carried out after 1 month.

### 2.3 Scale‐up experiment

The experiments conducted at laboratory level and with the use of pasteurized must were followed by the scale‐up trials. The goal was to evaluate the previously obtained results in real winemaking conditions which do not imply the pasteurization of the must. For this purpose, experiments were carried out in 200‐liter stainless still tanks. Grape processing and wine fermentation were conducted in an identical way as in the case of the laboratory experiment. The only difference was the use of fresh unpasteurized grape must. After the end of fermentation, the content of ethanol, glycerol, and volatile acids was determined.

### 2.4 Analyses

Total sugars, total acidity (expressed as tartaric acid), volatile acidity (expressed as acetic acid), and ethanol content (hydrostatic balance Densi Alcomat; Gibertini) of the must and produced wines were determined using official OIV methods (OIV, 2016). The content of yeast assimilable nitrogen in the must was determined by formol titration method (Zoecklein, Fugelsang, Gump, & Nury, 1999). The content of glycerol was determined by a commercial enzyme test (Megazyme, CO).

Concentration of methanol, ethanol, and higher alcohols was determined by gas chromatographic analysis using gas chromatograph Agilent 7890A equipped with flame ionization detector (FID) and a split/splitless injector, while a capillary column HP‐INNOWax (polyethylene glycol; 30 m × 250 µm i.d. with 0.25 µm film thickness) was used. The GC‐FID conditions were previously described (Miljić, Puškaš, Vučurović, & Muzalevski, 2017).

### 2.5 Wine sensory evaluation

Buxbaum model of positive ranking, described in the paper of Amerine and Roessler (1983), was applied for the evaluation of sensory properties of experimental wines. Sensory evaluation was performed by a panel of five qualified testers (officially certified tasters authorized for wine sensory analysis by Serbian Ministry of Agriculture). Four sensorial experiences rated up making a maximum of 20 points (up to 2 points for color, up to 2 points for clearness, up to 4 points for aroma, and up to 12 points for overall flavor). Overall flavor implies both taste and aroma components evaluated by retronasal olfaction. The minor unit of the scale is 0.1. The better the parameter is rated, the higher the mark is given. The bottom part of the evaluation sheet contained the space for tasters' comments since they were asked to highlight the most dominant aroma descriptors of the assessed wines.

Wines were presented to panelists in ISO standard wine glasses, in isolated booths, and under daylight‐type lighting. All wine samples were evaluated by the panel (seven trial sets, Table 1) with randomized presentation order. Three sessions were employed in three consecutive days where the panelist evaluated all seven individual wines each day in two sets. Each replicate presented on three consecutive days of tasting was poured from a separate bottle.

### 2.6 Statistical analysis

Statistical analysis in the present study was performed using Statistica 12.0 (StatSoft). The statistical difference between mean values of parameters was estimated by analyses of variance (ANOVA), at the 95% confidence level. Values detected as significantly different by the use of Duncan multiple range test were marked with different letters (a, b, c …).

## 3 RESULTS AND DISCUSSION

### 3.1 The influence of different yeast strains’ metabolic activity on ethanol production

Different selected yeast strains were used in wine fermentation trials, and the effect of their metabolic activity on the content of most important fermentation products was assessed. At the same time, the activity of autochthonous microbiota was suppressed by pasteurization of the must.

**Figure 1:**
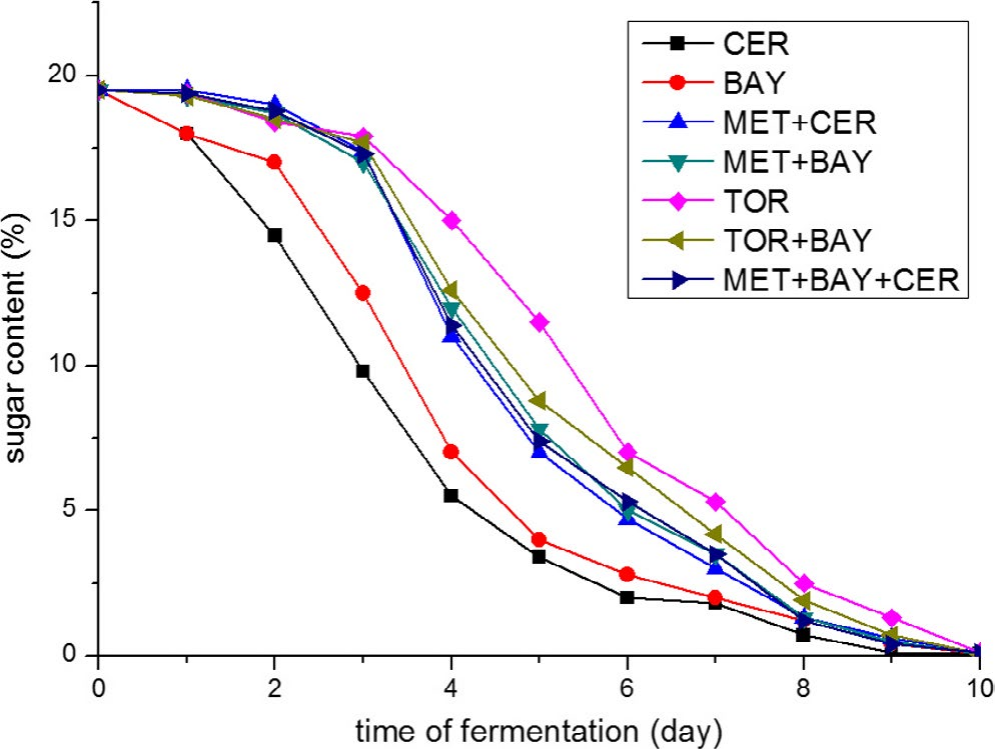
Sugar consumption profiles during the alcoholic fermentation of pasteurized must inoculated with different yeast strains in laboratory conditions. Marks describing each trial set are given in the figure legend: *Saccharomyces cerevisiae—*CER; *Saccharomyces bayanus—*BAY; *Torulaspora delbrueckii—*TOR; *Metschnikowia pulcherrima—*MET; a detailed inoculation plan is given in Table 1

Sugar consumption profiles during the fermentation of must inoculated according to the defined inoculation plan are shown in Figure 1. A different rate of sugar consumption between individual and sequential inoculation regime was observed. The use of individual yeasts *S. cerevisiae* and *S. bayanus* resulted in the shortest time of fermentation, that is, the highest rate of sugar consumption. Also, the sugars were almost consumed completely in 6 days of fermentation by these individual yeast strains. The activity of *M. pulcherrima* in the first 48–72 hr of fermentation, before the inoculation with *S. cerevisiae* and *S. bayanus*, resulted in a slight decrease in sugar content. After sequential inoculation, *M. pulcherrima* ferments (MET+CER, MET+BAY, and MET+CER+BAY) completed fermentation in 8–9 days. *T. delbrueckii* showed similar fermentation activity as *M. pulcherrima* with the slower sugar consumption in the first 3 days of fermentation. These results may be the consequence of a higher sensitivity to SO_2_ of *M. pulcherrima* and *T. delbrueckii*. Higher sensitivity to SO_2_ increases the time necessary for yeasts to adapt to the environmental conditions and to start the alcoholic fermentation, which is associated with the reduced consumption of sugars. In the end, it is important to emphasize that all treatments produced wines that had 0.5–1.0 g/L of reducing sugars.

**Figure 2:**
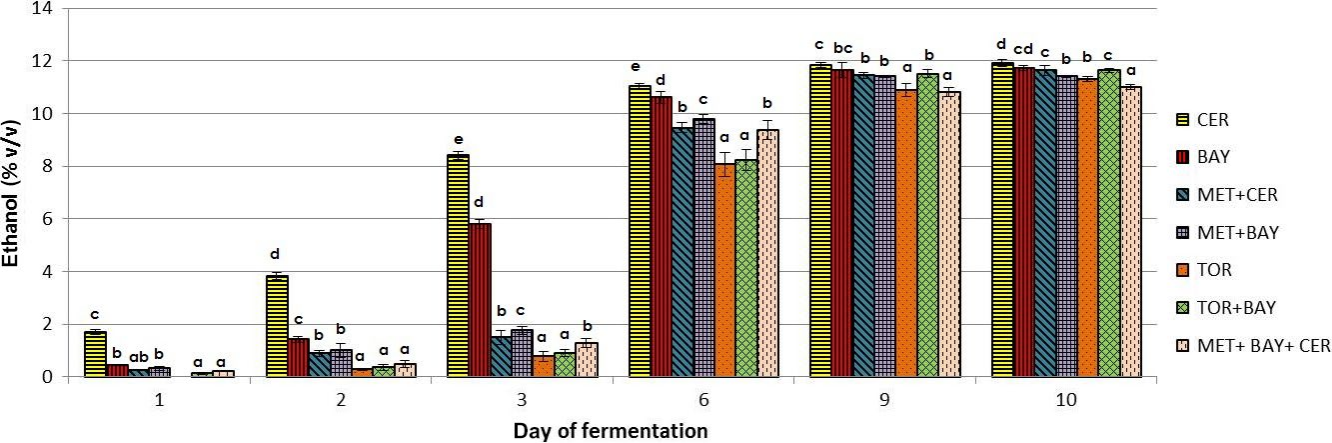
Changes in ethanol content during alcoholic fermentations carried out by different commercial yeast strains used in laboratory conditions. Marks describing each trial set are given in the figure legend: *Saccharomyces cerevisiae—*CER; *Saccharomyces bayanus—*BAY; *Torulaspora delbrueckii—*TOR; *Metschnikowia pulcherrima—*MET; a detailed inoculation plan is given in Table 1.^a,b,c^ different letters for the results in every specific day of alcoholic fermentation indicate significant differences between values (*p* < .05)

**Figure 3:**
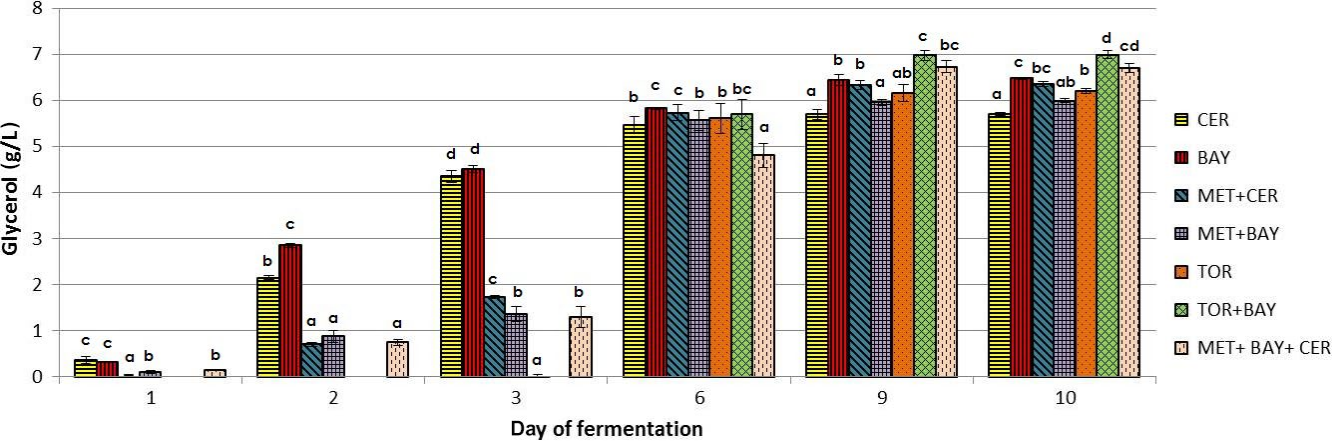
Changes in glycerol content during alcoholic fermentations carried out by different commercial yeast strains used in laboratory conditions. Marks describing each trial set are given in the figure legend: *Saccharomyces cerevisiae—*CER; *Saccharomyces bayanus—*BAY; *Torulaspora delbrueckii—*TOR; *Metschnikowia pulcherrima—*MET; a detailed inoculation plan is given in Table 1. ^a,b,c^ different letters for the results in every specific day of alcoholic fermentation indicate significant differences between values (*p* < .05)

**Figure 4:**
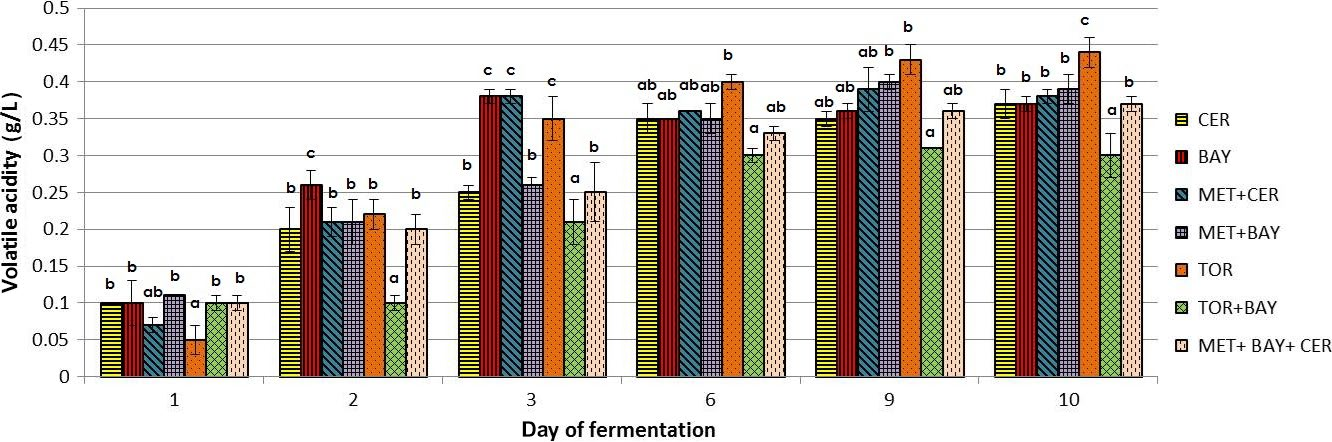
Changes in volatile acidity during alcoholic fermentations carried out by different commercial yeast strains used in laboratory conditions. Marks describing each trial set are given in the figure legend: *Saccharomyces cerevisiae—*CER; *Saccharomyces bayanus—*BAY; *Torulaspora delbrueckii—*TOR; *Metschnikowia pulcherrima—*MET; A detailed inoculation plan is given in Table 1. ^a,b,c^ different letters for the results in every specific day of alcoholic fermentation indicate significant differences between values (*p* < .05)

Figures 2, 3, 4 show changes in the content of the observed components (concentration of ethanol and glycerol and volatile acidity value) during the alcoholic fermentation of experimental wines. Through the comparison of the final values of observed parameters, the efficiency of applied yeast strains in the production of wines with reduced ethanol concentration was assessed.

The highest content of ethanol (11.92% v/v) was determined in the experimental wine produced only using a commercial strain of *S. cerevisiae*, while sequential inoculation of grape must with *M. pulcherrima, S. bayanus,* and *S. cerevisiae* resulted in the production of the lowest amount of this compound in the test wines (11.01% v/v). A significant reduction in the production of ethanol is also registered in the experimental wines produced by the activity of *M. pulcherrima* and *S. bayanus* (MET+BAY) and *T. delbrueckii* with the final ethanol concentration of 11.30% v/v and 11.40% v/v, respectively (Figure 2).

The highest rate of ethanol production (fermentative power) was observed in wines produced only with *S. cerevisiae*, where approximately 70% of the ethanol content formed within the first 3 days of fermentation. Fermentation of grape must inoculated only with *S. bayanus* showed the lower fermentative power (about 50% of the total ethanol) in the first 3 days compared with fermentation using a pure culture of *S. cerevisiae*.

In the grape must fermented with *M. pulcherrima,* the ethanol concentration in the first 3 days of fermentation was only 1.30%–1.70% v/v, while it is assumed that the rest of the formed alcohol is produced mainly by the activity of *S. bayanus* and *S. cerevisiae* after sequential inoculation. The activity of *T. delbrueckii* resulted in an ethanol concentration of 0.8%–0.9% v/v in the early days of fermentation. The highest ethanol production rate with a pure culture of *T. delbrueckii* was achieved between the third and sixth days of fermentation.

Previous studies (Canonico, Comitini, Oro, & Ciani, 2016; Contreras, Curtin, & Varela, 2015; Contreras et al., 2014; Gobbi et al., 2013; Varela, Barker, Tran, Borneman, & Curtin, 2017) have reported a significant reduction in ethanol yield (0.3%–1.7% v/v) when using non‐*Saccharomyces* and *S. cerevisiae* strains in co‐inoculated or sequential cultures. The use of *M. pulcherrima* co‐inoculated with *S. cerevisiae* in the pilot‐scale production of Merlot wines resulted in 1.0% v/v lower ethanol content compared to wines fermented by *S. cerevisiae* alone (Varela et al., 2017).

Contreras et al. (2014) have reported that sequential inoculation of the selected strain of *M. pulcherrima* AWRI1149 and *S. cerevisiae*, after 3 days, resulted in the production of wines with a lower concentration of ethanol in relation to wine produced only by the application of *S. cerevisiae* (in Chardonnay, the decrease was 0.9% v/v, while in Shiraz, this reduction was 1.60% v/v). Canonico et al. (2016) investigated the use of immobilized selected strains of non‐*Saccharomyces* yeasts (*Starmerella bombicola*, *M. pulcherrima*, *Hanseniaspora osmophila*, and *Hanseniaspora uvarum*) to start fermentation, followed by inoculation of free *S. cerevisiae* cells. The sequential inoculations of *S. bombicola‐* and *M. pulcherrima*‐immobilized cells and *S. cerevisiae*‐free cells showed the best reductions in the final ethanol content (1.6% and 1.4% v/v, respectively). Also, lower ethanol production was recorded in ferments obtained by joint activity of different *Saccharomyces* species. Sequential inoculation of *Saccharomyces uvarum* (AWRI 2846) and *S. cerevisiae* resulted in ethanol reduction of 0.8% v/v and an increase in glycerol content for 6.4 g/L (Contreras, Curtin, et al., 2015). Moreover, wines fermented with *S. uvarum* in the study of Varela et al. (2017) had a 1.7% v/v lower ethanol concentration than *S. cerevisiae* ferment. Previous works also showed that the application of non‐*Saccharomyces* yeast in fermentations with controlled aeration can be an efficient approach for the production of wines with decreased alcohol content (Canonico, Comitini, & Ciani, 2019; Canonico, Solomon, Comitini, Ciani, & Varela, 2019; Contreras, Hidalgo, et al., 2015). The following experiments generally implied controlled aeration (1–10 ml L^‐1^ min^‐1^) during the first 24–72 hr of fermentation. Under these conditions, *M. pulcherrima*, *T. delbrueckii,* and *Z. bailii* strains produced wines with 0.9%–2.0% v/v lower ethanol content compared to *S. cerevisiae* wines.

Glycerol is nonvolatile three‐hydroxy alcohol which indirectly contributes to the sensory character of a wine. At high concentration, it contributes significantly to the sweetness, body, and fullness of wines. For these reasons, glycerol production is one of the desirable features during grape must fermentation. Furthermore, of all the microbiological strategies to reduce ethanol yield, glycerol overproduction was proven to be the most effective (Varela et al., 2012). Among experimental wines produced in this study, the highest glycerol content (6.99 g/L) was obtained during sequential fermentation with *T. delbrueckii* and *S. bayanus* (Figure 3). Sequential inoculation of strain *S. bayanus, M. pulcherrima*, and *S. cerevisiae* also resulted in relatively high value (6.7 g/L) of glycerol. The lowest production of this compound (5.7 g/L) was established in the wine produced only with *S. cerevisiae*. Similar results for glycerol levels were reported by the study of Canonico et al. (2016). From the obtained results, it can be noticed that *S. bayanus*, among the tested yeasts, was the most efficient glycerol producer. The individual inoculation with both *S. cerevisiae* and *S. bayanus* led to an intensive glycerol production in the first 3 days of fermentation which resulted in formation of more than 70% of total glycerol amount in this period. Available literature (Ribéreau‐Gayon, Dubourdieu, Donèche, & Lonvaud, 2006) points out that the production of glycerol is associated with fermentation of the first 50 g/L of sugar from must, and these data relate primarily on *S. cerevisiae* yeast activity. From the obtained results, it can be noticed that the production of glycerol in the ferments with *T. delbrueckii* during the first 3 days was minimal. *T. delbrueckii* was shown to be a lower producer of secondary by‐products of fermentation (Ciani et al., 2016). Glycerol production by *M. pulcherrima* in the first 3 days of fermentation was also low (1.3–1.75 g/L), compared to the individual fermentations by *S. cerevisiae* and *S. bayanus*. Positive linear correlation between the production of ethanol and glycerol was recorded in fermentations started by *S. cerevisiae*, *S. bayanus,* and *M. pulcherrima* (0.991, 0.918, and 0.933, respectively). On the other hand, significantly lower degree of linear correlation (.516) was determined between ethanol and glycerol content in *T. delbrueckii* ferments.

Volatile acidity (VA) is a parameter which significantly determines the quality of wine, and, in general, it represents the content of acetic acid as a by‐product of alcoholic fermentation (more than 80% of VA originates from acetic acid). If adequate vinification practices had been employed, the resulting values of this parameter are below the ones that can negatively impact the organoleptic properties of the wine. According to Zoecklein et al. (1999), the sensory detection threshold of volatile acidity in the wine is in the range of 0.7–1.1 g/L. Analysis of produced experimental wines (Figure 4) showed the highest volatile acidity in wines obtained by the activity of *T. delbrueckii* (0.44 g/L), but similar values were determined also for the other ferments (ranging 0.30–0.40 g/L). It should be emphasized that higher glycerol production in some ferments (TOR+BAY and MET+BAY+CER) was not followed by the increase in volatile acidity. This is very important, especially from the organoleptic point of view. The changes of the volatile acidity recorded during fermentations with individual or mixed yeast cultures clearly indicate that the most intensive production of these compounds is in the first 3 days in all fermentations.

### 3.2 The impact of used yeast strains on the composition of volatile compounds in wines

The aroma is one of the main characteristics that determine the quality and value of a wine. The aroma of wine is a unique mixture of volatile compounds originating from grape and secondary products formed during the wine fermentation and aging. A large number of volatile compounds are formed by yeast during alcoholic fermentation, and they significantly impact the aroma and overall quality of wines. The most important volatile compounds synthesized by wine yeast include higher alcohols, acetate esters, ethyl esters, and aldehydes among others. The influence of commercial selected yeast strains used individual or sequential inoculation, on the composition of the aromatic compounds in experimental wines, is shown in Table 2.

**Table 2:** Concentration of the major volatile compounds in wines produced in laboratory‐scale experiment

Volatile compound (mg/L)	CER	BAY	MET+CER	MET+BAY	TOR	TOR+BAY	MET+BAY+CER
Acetaldehyde	6.5 ± 0.3^a^	13 ± 1.2^bc^	18 ± 0.9^c^	7.4 ± 0.7^a^	10.7 ± 0.8^b^	7 ± 0^a^	15 ± 0.5^c^
Ethyl acetate	49 ± 1.5^a^	49 ± 0.7^a^	48.2 ± 2.3^a^	49.4 ± 1.9^a^	46.1 ± 1.1^a^	49.8 ± 1.5^a^	47.5 ± 1.1^a^
2‐Butanol	nd	nd	nd	nd	nd	nd	nd
2‐Propanol	nd	nd	nd	nd	nd	nd	nd
1‐Propanol	0.7 ± 0.1^a^	4.5 ± 0.4^b^	1.0 ± 0.2^a^	1.1 ± 0^a^	0.4 ± 0.1^a^	3.7 ± 0.3^b^	0.9 ± 0^a^
2‐Methyl−1‐propanol	11.7 ± 0.8^a^	11.6 ± 0.7^a^	12.2 ± 1.2^a^	11.6 ± 1.4^a^	10.9 ± 0.3^a^	11.5 ± 0.7^a^	11.4 ± 0.8^a^
1‐Butanol	nd	nd	nd	nd	nd	nd	nd
3‐Methyl−1‐butanol	26.4 ± 2.4^b^	23.9 ± 1.3^b^	28.1 ± 0.7^c^	22.8 ± 1^b^	20.6 ± 1.5^ab^	18.9 ± 0.6^a^	24 ± 1.5^b^
1‐Pentanol	nd	nd	nd	nd	nd	nd	nd
1‐Hexanol	nd	nd	nd	nd	nd	nd	nd
1‐Heptanol	10.7 ± 0.1^a^	23 ± 1.9^d^	19.5 ± 1.0^c^	14.5 ± 0.5^b^	19.7 ± 1^c^	9 ± 0.8^a^	16.4 ± 0.4^bc^
Furfural	0.6 ± 0.2^a^	0.7 ± 0.2^a^	0.8 ± 0.3^a^	0.6 ± 0.2^a^	1.1 ± 0.3^a^	0.9 ± 0.3^a^	0.4 ± 0.1^a^
Benzaldehyde	48.5 ± 2.1^a^	76.4 ± 1^d^	49.5 ± 0.7^a^	56.8 ± 1.4^b^	61.2 ± 1.5^c^	87.3 ± 1.9^e^	51.4 ± 0.9^a^
1‐Octanol	22 ± 1.2^a^	23.2 ± 1^a^	21.7 ± 1.5^a^	21.5 ± 0.8^a^	21.2 ± 0.3^a^	23.5 ± 1.2^a^	20.5 ± 0.8^a^
1‐Nonanol	5.2 ± 0.4^a^	nd	nd	5.4 ± 0.7^a^	5.1 ± 0.4^a^	nd	nd
1‐Decanol	6.2 ± 0.2^a^	5.9 ± 0.6^a^	5.7 ± 0^a^	6.3 ± 0.8^a^	6.7 ± 0.2^a^	5.9 ± 0.7^a^	5.4 ± 0.2^a^
Benzyl alcohol	10.5 ± 0.6^c^	8 ± 0.4^b^	11.2 ± 0.8^c^	9.1 ± 2^b^	8 ± 0.7^b^	5.8 ± 0.3^a^	9.7 ± 0.8^bc^

Note

*Saccharomyces cerevisiae—* **CER**; *Saccharomyces bayanus—* **BAY**; *Torulaspora delbrueckii—* **TOR**; *Metschnikowia pulcherrima—* **MET**. Different letters in the same row indicate significant differences between values (*p* < .05). nd, not detected.

Metabolic activity of commercial selected yeast strains during fermentation caused significant differences in the content of aromatic compounds in produced wines. As shown, ten higher alcohols and two aldehydes were detected in the analyzed wine samples; meanwhile, the compounds such as 2‐propanol, 1‐butanol, 2‐butanol, 1‐pentanol, and 1‐hexanol were not detected. The produced wines were characterized by relatively uniform content of ethyl acetate, 2‐methyl‐1‐propanol, that is, isobutanol, octanol, and decanol. The use of commercial non‐*Saccharomyces* strains did not lead to the increase in the ethyl acetate levels, and the amounts determined (about 50 mg/L) were far below the threshold associated with a negative impact on wine sensory characteristics (above 150 mg/L). The content of acetaldehyde was the highest in the MET+CER and MET+BAY+CER samples (15–20 mg/L); however, even the highest levels determined were significantly lower than the ones associated with oxidative faults (“over‐ripe bruised apples,” “sherry,” and “nut‐like” characters). At lower levels (below 50 mg/L), acetaldehyde can contribute pleasant fruity aromas to a wine. As for comparison, Varela et al. (2017) and Canonico et al. (2016) reported that wines produced with *M. pulcherrima* showed higher concentrations of ethyl acetate, total esters, total higher alcohols, and total sulfur compounds compared to *S. cerevisiae* ferments. The wine obtained by *S. bayanus* activity had a significantly higher concentration of 1‐propanol (samples BAY and TOR+BAY) and 1‐heptanol (sample BAY) compared to the rest of the experimental wines. Also, the wines BAY and TOR+BAY had significantly higher amounts of benzaldehyde (76.4 and 87.3 mg/L, respectively), in comparison with other experimental wines. The highest contents of 3‐methyl‐1‐butanol (24–28 mg/L) and benzyl alcohol (9.7–11.2 mg/L) were detected in wines produced by the activity of *S. cerevisiae*, in both individual and sequential inoculations (samples CER, MET+CER, and MET+BAY+CER). The presence of furfural was detected in small amounts in all wine samples. One of the main sources of furfural in wines is toasted oak wood. Considering the fact that wines in this study were not in contact with wood, the possible explanation for furfural occurrence is the pasteurization of must before inoculation of yeast strains.

### 3.3 Wine sensory analysis

The results of the sensory analysis (Table 3) indicate that a difference in the aroma, as well as taste and flavor, was recorded in experimental wines produced using different commercially available yeast strains in laboratory conditions. On the other hand, the color differences could be considered insignificant. In terms of aroma, the best‐evaluated wines were produced by the activity of yeasts MET+BAY and TOR+BAY. The lowest marks were given to MET+CER and MET+BAY+CER wines, which can be due to the higher content of acetaldehyde compared to other wines. Wine MET+BAY was characterized by citrus flavors, while in MET+CER, melon and banana flavor dominated. Wines MET+BAY and TOR+BAY had fuller, more complex, and rounded flavors compared to other experimental wines. These properties could partly be impacted by the higher contents of 1‐propanal, 1‐heptanol (flower tones), and benzaldehyde (almond flavor) compared to other wines. The use of *M. pulcherrima* and *T. delbrueckii* in co‐inoculation with *S. bayanus* commercial strains gave the complexity to the sensory profiles of produced wines which was confirmed by the highest taste and overall flavor marks, as well as the highest overall marks (19.2–19.3). Varela et al. (2017) used a consensus‐based descriptive methodology and demonstrated that apart from being able to produce wine with reduced alcohol concentration, *M. pulcherrima* in co‐inoculation with *S. cerevisiae* gave wines with similar sensory profiles (not differing significantly in any attribute and with high scores given for desirable sensory descriptors) as uninoculated and control *S. cerevisiae* ferments.

**Table 3:** Sensory evaluation of wines produced in laboratory‐scale experiment

	Color (max 2 points)	Clearness (max 2 points)	Aroma (max 4 points)	Overall flavor (max 12 points)	Total (max 20 points)
CER	2.0 ± 0.0^a^	2.0 ± 0.0^a^	3.2 ± 0.2^a^	11.3 ± 0.2^ab^	18.5 ± 0.2^a^
BAY	2.0 ± 0.0^a^	2.0 ± 0.0^a^	3.6 ± 0.1^b^	11.4 ± 0.0^b^	19.0 ± 0.1^bc^
MET+CER	2.0 ± 0.0^a^	2.0 ± 0.0^a^	3.2 ± 0.1^a^	11.2 ± 0.1^a^	18.4 ± 0.1^a^
MET+BAY	2.0 ± 0.0^a^	2.0 ± 0.0^a^	3.7 ± 0.0^b^	11.5 ± 0.1^bc^	19.2 ± 0.1^c^
TOR	2.0 ± 0.0^a^	2.0 ± 0.0^a^	3.4 ± 0.1^a^	11.4 ± 0.2^b^	18.8 ± 0.2^b^
TOR+BAY	2.0 ± 0.0^a^	2.0 ± 0.0^a^	3.7 ± 0.1^b^	11.6 ± 0.0^c^	19.3 ± 0.1^c^
MET+BAY+CER	2.0 ± 0.0^a^	2.0 ± 0.0^a^	3.4 ± 0.2^a^	11.0 ± 0.2^a^	18.4 ± 0.2^a^

Note

The data represent the average values of the scores given by five members of the panel. Different letters in the same column indicate significant differences between values (*p* < .05).

### 3.4 Scale‐up experiment

**Figure 5:**
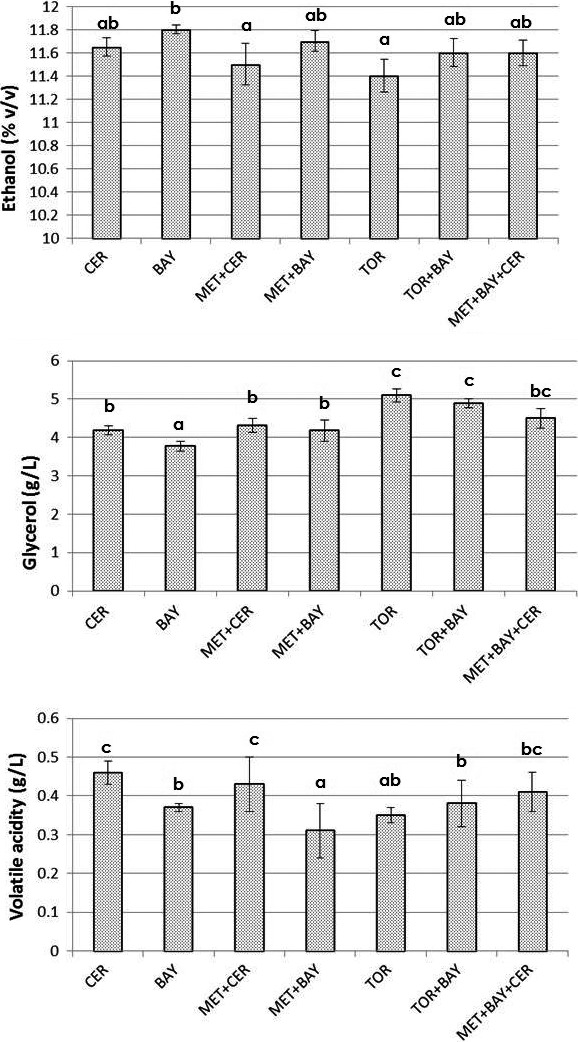
Final concentrations of glycerol, volatile acids, and ethanol in wines produced by different commercial yeast strains used in scale‐up conditions. Marks describing each trial in the figure legend: *Saccharomyces cerevisiae—*CER; *Saccharomyces bayanus—*BAY; *Torulaspora delbrueckii—*TOR; *Metschnikowia pulcherrima—*MET; a detailed inoculation plan is given in Table 1. ^a,b,c^ different letters indicate significant differences between values (*p* < .05)

Evaluation of the results obtained in the laboratory experiments was done by simulating the same vinification conditions in the scale‐up experiment. The aim was to assess the activity of applied yeast strains in a real must sample and in the presence of autochthonous microbiota. The final concentration of ethanol and glycerol, and value of volatile acidity in these wines are shown in Figure 5. The aptitude of commercial yeast strains for lowering the ethanol content of wine was lower compared to ferments based on sterile must use. The competitiveness between inoculated non‐*Saccharomyces* yeasts and yeasts from autochthonous microbiota resulted in a slower start of fermentation than in the laboratory experiment (results not shown). Moreover, the content of ethanol was in a pretty narrow range 11.4%–11.8% v/v, while the content of glycerol was 3.78–4.89 g/L which is significantly lower than that obtained in wines from laboratory‐scale experiment (5.7–6.99 g/L). The value of volatile acidity was very similar in both experimental sets (0.3–0.45 g/L). Similar differences among the results of experiments with model and real must samples were reported (Ciani & Ferraro, 1998; Ferraro, Fatichenti, & Ciani, 2000). The use of *Starmerella bombicola* (formerly *C. stellata*) and *S. cerevisiae* resulted in a high production of glycerol, succinic acid, and different by‐products (interactions involving acetaldehyde and acetoin) with a consequent reduction of final ethanol amount. The reduction in ethanol content in these experiments varied from 0.64% v/v at pilot scale in natural grape juice to 1.60% v/v at laboratory scale using synthetic grape juice. Furthermore, different factors affecting the metabolism of *M. pulcherrima* AWRI1149 during fermentation of nonsterile Shiraz must were evaluated (Contreras, Curtin, et al., 2015). Among different inoculation regimes which were applied, only initial inoculation with 1 × 10^6^ cells/mL of *M. pulcherrima* AWRI1149, followed by *S. cerevisiae* after 50% sugar consumption, leads to a significant ethanol concentration reduction. Canonico et al. (2016) showed that application of immobilized selected strains of *M. pulcherrima*, followed by inoculation of free *S. cerevisiae* cells, resulted in a decrease in ethanol content for 1.3% v/v when synthetic grape juice was used, while in the case of natural grape juice the reduction was 1% v/v (in both trials, the beads of immobilized yeast were removed after 72 hr from inoculation).

## 4 CONCLUSION

In summary, this work evaluated the potential of commercial selected yeast strain application for purpose of wines with decreased alcohol content production. Sequential inoculation of the must with *M. pulcherrima*, *S. bayanus,* and *S. cerevisiae* resulted in the production of wines with the lowest ethanol content among experimental samples (decrease of 0.9% v/v compared to the control wine). Significant differences in the content of certain aromatic compounds, as well as in taste and flavor, were also found in produced wines. The experiment in real conditions showed that used commercially available *Saccharomyces* and non‐*Saccharomyces* were not effective enough in lowering ethanol concentration in wines due to interactions and competitiveness with yeasts from autochthonous microbiota. Already evident problems of wines with very high alcohol content will force wine producers in near future to find more efficient ways of directing alcoholic fermentation to the production of wines with lower alcohol content and not worsened sensory properties. The application of *Saccharomyces* and non‐*Saccharomyces* strains obtained by adaptive evolution principles (non‐GM) for this purpose will need further investigation and commercialization.

## CONFLICT OF INTEREST

The authors declare that they have no conflicts of interest.

## ETHICAL APPROVAL

This study does not involve any human or animal testing.
